# Relevance of Anatomical Significance of AV Nodal Structures within Koch’s Triangle and Pyramid

**DOI:** 10.3390/jcdd11100323

**Published:** 2024-10-14

**Authors:** Andrea Matteucci, Claudio Pandozi, Maurizio Russo, Marco Galeazzi, Giammarco Schiaffini, Marco Valerio Mariani, Carlo Lavalle, Furio Colivicchi

**Affiliations:** 1 Clinical and Rehabilitation Cardiology Division, San Filippo Neri Hospital, 00135 Rome, Italy; 2 Department of Experimental Medicine, Tor Vergata University, 00133 Rome, Italy; 3 Department of Cardiovascular, Respiratory, Nephrological, Anesthesiological and Geriatric Sciences, “Sapienza” University of Rome, 00166 Rome, Italy

**Keywords:** cardiac conduction system, AV node, ablation, AVNRT, integrated diagnostic approaches

## Abstract

The exploration of the cardiac conduction system evolved over a century, marked by groundbreaking discoveries in atrioventricular (AV) nodal physiology. Atrioventricular nodal re-entrant tachycardia (AVNRT), the most prevalent regular tachycardia in humans, remains enigmatic despite extensive research. Detailed examinations of AV nodal anatomy and histology reveal variations in location and shape, influencing electrophysiological properties. Variability in AV nodal extensions and their embryological origins contribute to the complexity of the conduction system. Physiologically, the AV node plays a crucial role in modulating AV conduction, introducing delays for ventricular filling and filtering atrial impulses. Dual-pathway physiology involving fast and slow pathways further complicates AVNRT circuitry. Integrated approaches combining pre-procedural imaging with electroanatomical mapping enhance our understanding of AV nodal structures and high-definition mapping improves precision in identifying ablation targets. Electrophysiological–anatomical correlations may unveil the specific roles of conduction axis components, aiding in the optimization of ablation strategies. This review traces the historical journey from Tawara’s pioneering work to recent integrated approaches aimed at unraveling the intricacies of AV nodal structures while emphasizing the importance of a multidimensional approach, incorporating technological advancements, anatomical understanding, and clinical validation in human mapping studies.

## 1. Introduction

The history of the cardiac conduction system spans over a century of dedicated scientific inquiry, beginning with Sunao Tawara’s groundbreaking work in 1906 [1] and the discovery of the atrioventricular (AV) node. Atrioventricular nodal re-entrant tachycardia (AVNRT) is nowadays the most prevalent regular tachycardia in humans, which is intricately linked to the anatomy and physiology of the AV nodal and junctional area. Despite extensive investigations, uncertainties persist regarding the exact mechanism, anatomical site, and circuit pathways sustaining AVNRT. Since Tawara’s pioneering work, advancements in anatomy, embryology, physiology, molecular biology, and the electrophysiological mapping of the atrioventricular junction have unfolded. Recent insights have witnessed new integrated approaches aimed at enhancing the exploration of atrioventricular nodal structures. In this review, we delve into a century of discoveries up to the latest new approaches being tested, exploring their implications for understanding the intricate anatomical organization of the AV node, to understand their importance in arrhythmias and cardiac physiology.

## 2. AV Node Anatomy and Histology

Understanding the electroanatomical mechanisms governing wall activation in the small area where the AV nodal structures reside is complex [2]. Defining the boundaries of these elements, even with three-dimensional mapping systems, presents difficulties. Recent studies focused on atrial inputs to the AV node due to the inability to electrically locate it [3]. The latest advances, using a mapping system and small electrode basket catheter, identified potentials from the compact node and its extensions [4]. Despite progress, visualizing the atrioventricular conduction axis with standard imaging remains impossible. Predictions based on anatomical landmarks and known dimensions are subject to variability. To enhance ablation procedure effectiveness and safety in the septal, paraseptal, and parahisian regions, optimal anatomical knowledge and integrated three-dimensional electroanatomic maps with computed tomographic preprocedural images are useful. Based on histology and immunolabeling in both animals and humans, the normal AV junctional area comprises distinct structures, including transitional tissue, inferior nodal extensions, the compact node, lower nodal bundle (LNB), and His bundle. The term AV node generally refers to the compact node. The classic so-called Koch’s triangle, traditionally defined by the fibrous tendon of Todaro, tricuspid valve leaflet attachment, the ostium of the coronary sinus, and septal isthmus, outlines the atrioventricular septum. However, recent studies propose redefining it as Koch’s pyramid, due to its relationship with the pyramid space and infero-septal recess, challenging traditional notions [3]. Variability exists in the compact AV node’s location within the atrioventricular junction, affecting electrophysiological properties [4]. Anderson et al. have reported that the compact AV node can be carried either on a fibrous plate at the apex of the inferior pyramidal space or be supported on the rightward surface of an area of fibrous continuity between the leaflets of the tricuspid and mitral valves [5]. Comparative anatomy studies have highlighted how the AV node exhibits different shapes, such as a triangle, ellipse, fan, or star, with dimensions ranging from 5 to 7 mm in length and 1.0–1.5 mm in width [6]. The His bundle continuation is marked by the lower border of the membranous septum and inter-leaflet fibrous triangle between the right and non-coronary leaflets of the aortic valve. Insights into the AVN’s anatomy include the discovery of inferior extensions (INEs), initially described by Tawara but later reevaluated by Hucker [7], Inoue and Becker [8]. INE, especially the right inferior extension (RINE), is considered a substrate for the slow pathway (SP) in humans [9]. In addition, rings of conduction tissue take their origin from the INE of the AV node, passing rightward and leftward to encircle the orifices of the tricuspid and mitral valves and reuniting to form an extensive retroaortic node [10]. Superior anatomical connections to the AVN involve myocardial cells linked to transitional cells on the atrial septum, serving as potential substrates for the fast pathway (FP) [11]. The SP generally traverses the area between the coronary sinus and the tricuspid annulus. It has a longer conduction time but a shorter effective refractory period. In contrast, the FP typically runs supreme, originating from the interatrial septum, and it has a quicker conduction rate but a longer effective refractory period [9]. During normal sinus rhythm, conduction occurs via the FP. However, at elevated heart rates or with premature beats, the SP often takes over, as the FP may be refractory under these conditions.

**Figure 1:**
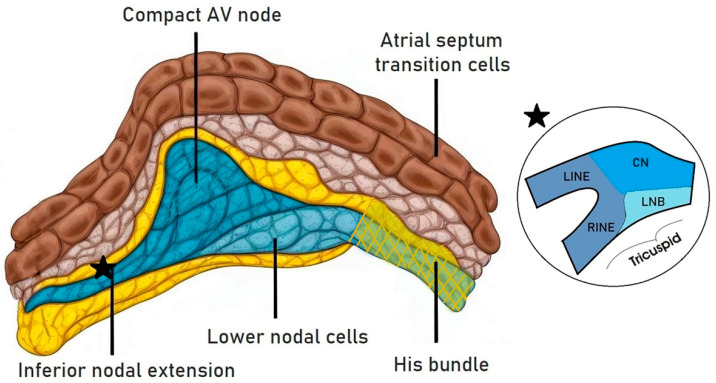
A representation of the different components of the atrioventricular junction. The lower nodal cells share a common origin with the His bundle cells. The black star reports the passage between compact node (CN), lower nodal bundle (LNB), the left and right inferior nodal extensions (LINE and RINE).

Variability exists in studies regarding the presence of the LNB in humans, with animal models like rabbit hearts showing a continuous bundle connected to RINE, the compact AV node, and the AV bundle [12]. Studies indicate substantial differences in the embryological origin, connexin types, and ionic currents involved in the structures of the AV conduction system, influencing their electrophysiological properties [13] (Figure 1).

**Figure 2:**
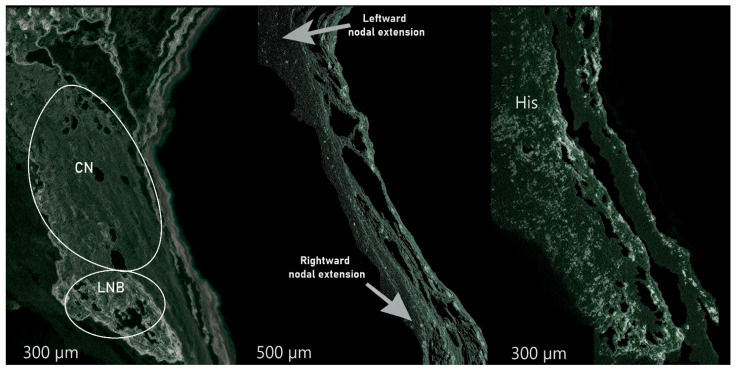
Cx43 density in the AV nodal structures. On the left, the compact AV node, and the lower nodal bundle; in the center, the left and right nodal extensions at the top and bottom, respectively; and finally, on the right, the His region. CN = compact node. LNB = lower nodal bundle.

Embryologically, the AV conduction system develops from a primary myocardium, with repression factors TBX2 and TBX3 influencing the atrioventricular canal formation [14]. The development of the AV conduction axis involves the primary ring, atrioventricular canal, and insulating tissues, contributing to the atrial and ventricular components. According to the findings of Anderson et al., the right inferior extension should retain specialized histological characteristics, capable of both slow and fast conduction, while the left inferior extension should be limited to slow conduction [15]. The speed of conduction in structures of the AV node depends on the expression of Na+ channels, cell diameter, and electrical coupling facilitated by connexins. In particular, connexins Cx40 and Cx43, which are primarily found in the atrial and ventricular myocardium, demonstrate diverse expression patterns within the AV node and its extensions, thereby impacting conduction velocity [16]. The bundle of His, as well as the LNB and bundle branches, express Cx43 and Cx40, while the compact node exhibits low Cx43 levels [17]. The Cx43 content in the RINE is still debated (Figure 2).

In addition, sodium channels express various isoforms in the human heart and AV node [18]. Nav1.5 is abundant in atrial and ventricular cells but minimal in the compact node (CN) and INE, influencing action potential characteristics [19]. Calcium channels, essential for AV node depolarization, involve Cav1.2 and Cav1.3, contributing to IcaL in CN and INE, impacting the action potential upstroke in these structures [20]. In summary, similar connexin and ion channel patterns in the AV node and its extensions indicate comparable conduction properties and shared embryological development. Contrastingly, LNB and PB exhibit parallel connexin staining and ion channel expression. Regional variations in electrical coupling, ionic currents, and action potential shape along the AV conduction axis result from distinct gap junction and ion channel expression.

## 3. AV Node Physiology

The AV node serves as a crucial element in modulating AV conduction, forming the proximal right atrial portion of the conduction axis [21]. It orchestrates AV synchrony by introducing a delay between atrial and His bundle activation during each heartbeat, enhancing ventricular filling in late diastole. Slow conduction through AV tissue, attributed to high membrane action potentials, low sodium channel accessibility, and abundant calcium current, contributes to this delay. However, this type of slow conduction is weak, because it occurs with a low safety factor for conduction that precludes the attainment of conduction velocity slower than 10 cm/s before conduction failure occurs, while conduction velocity in the AV node may be as slow as 2 cm/s [16]. Structural discontinuities due to cellular electrical uncoupling, because of the low density of gap junctions and reduced connexins’ expression, are the other and more important mechanisms playing a role in slow AV node conduction. Such structural discontinuities can support very slow conduction with a high safety factor, allowing for a conduction velocity as slow as 1 cm/s before the occurrence of the conduction block [22]. The AV node also filters atrial impulses, protecting the ventricles from excessive activation during atrial arrhythmias. The Wenckebach periodicity and concealed conduction during atrial fibrillation are notable mechanisms underlying this filtering action. Despite uncertainties surrounding the precise process, two hypotheses, the decremental propagation theory, and the functional step delay theory, attempt to explain the Wenckebach phenomenon [23,24]. Additionally, the AV nodal fibers exhibit slow diastolic depolarization, potentially leading to automatic impulse formation, acting as a subsidiary pacemaker when the sinoatrial node fails [25]. The AV junction displays dual-pathway physiology, involving FP and SP wavefronts propagating to the His bundle. Traditionally, longitudinally dissociated dual AV nodal pathways explained this phenomenon, and recent evidence identifies the right inferior extension as the SP and the septal connection as the fast FP [4]. Complex atrial inputs, including superior and inferior connections, sinus septum, and the left side of the atrial septum, contribute to dual-pathway physiology. Notably, SP ablation in patients with a long PR interval highlights the functional persistence of anterograde conduction along pathways other than the FP [26]. Moreover, radiofrequency ablation studies about AV conduction during atrial fibrillation in the SP area show changes in AV node effective refractory periods similar to those observed in AVNRT patients who undergo SP ablation, emphasizing the physiological nature of dual-pathway electrophysiology [27]. Detailed studies on the routes of fast retrograde and slow anterograde pathways reveal their complexity, challenging traditional models. Anatomical findings suggest that the most distal connection between the atrial septal myocardium and the AV node, constituting the FP in typical AVNRT, could be an atrio-hisian connection bypassing the entire AV node [8]. Furthermore, Hucker proposed new models with anterograde SP conduction involving the RINE spreading through the LNB to the His bundle, bypassing the compact AV node. This hypothesis is supported by electrophysiological studies on rabbits and optical mapping observations [28]. Fast and slow anterograde pathways exhibit characteristic changes in the amplitude and timing expression of dual-pathway conduction, as seen in the rabbit His bundle electrogram alternans, known as Zhang’s phenomenon [29]. For all these reasons, defining the precise anatomical location of Koch’s pyramid arrhythmias remains challenging. Early models posited longitudinally dissociated dual AV nodal pathways, but subsequent evidence challenges this simplicity. Recent proposals highlight the role of AV nodal extensions, connexin genotyping, and three-dimensional reconstruction to identify structures with different conduction properties within Koch’s pyramid [17]. Discordant results regarding Cx43 levels in the human RINE raise questions about its conduction properties [30]. The possibility of multiple inputs and the involvement of structures like the retroaortic node further complicate the circuitry hypothesis. Mathematical modeling and studies on humans and animals contribute to the ongoing effort to unravel the intricate circuitry of typical AVNRT. Finally, as a culmination of the latest findings, the exploration of AVNRT circuits has expanded to encompass the septal isthmus, the ring of conduction tissue, and the retroaortic node, providing valuable insights into the latest advancements in our understanding [31,32].

## 4. Integrated Approaches for a Precise Definition of the Anatomical Structures of Koch’s Pyramid

**Figure 3:**
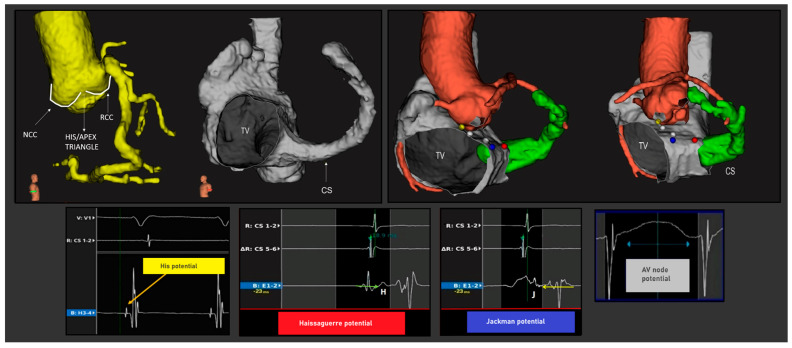
Merged acquisition from the 3D mapping and reconstruction of computed tomographic datasets, indicating different stages. Geometry import is available to import geometries created by another application (such as computed tomography or magnetic resonance imaging) and align it to the already created maps, such that maps can be compared to the imported geometry. The imported geometrical shells can be used as a reference, for example, to identify anatomical features in advance of mapping. In this example, a computed tomographic dataset for the segmentation of the aortic root and the coronary arteries was used, along with the right atrium including the coronary sinus. Once the electroanatomical map had been acquired, it was aligned with the reconstructed segmentations. This alignment allows us to identify the aortic root relative to Koch’s pyramid and correlated AV nodal structures and electrograms, listed as follows: His (yellow tag), AV node (white tag), Jackman potentials (red tag) and Haissaguerre potentials (blue tag) are shown. NCC = non coronary cuspid, RCC = right coronary cuspid, TV = tricuspid valve, CS = coronary sinus.

The integration of data derived from pre-procedural cardiac imaging along with electroanatomical mapping has the potential to identify novel ablation targets. In this perspective, a recent study showed how information from cardiac CT or cardiac MRI studies can be combined with electroanatomical maps to provide a greater definition of anatomical structures, which sometimes cannot be easily visualized, and to improve the accuracy and details of the maps obtained [33]. Several software programs facilitate the segmentation of cardiac chambers, aorta, and coronary arteries [34] and allow their integration into mapping systems to reconstruct the dimensions and the location of the components of the conduction axis, confirmed by the recording of specific potentials and endocavitary electrograms. This is even more useful when multi-polar catheters are used, allowing for high-definition mapping which can be integrated with a direct recording of AV node structure potential [35], reaching a more precise correlation with the merged radiological scans (Figure 3).

The knowledge of the compact node’s location, as indicated by specific electrogram characteristics, has the potential to enhance the safety and efficacy of radiofrequency ablation procedures [36]. This understanding could reshape ablation strategies for various conditions, optimizing outcomes and minimizing complications, and reducing, for example, arrhythmias associated with direct His bundle injury. Establishing an electrophysiological–anatomical correlation may unveil the specific roles of conduction axis components within Koch’s pyramid and triangle, linking electrophysiological signatures to anatomical substrates. Such correlations may identify potential targets for the ablation of arrhythmias originating in parahisian septal and paraseptal atrial or ventricular foci, or sustained by accessory pathways traversing the parahisian area.

## 5. Parahisian Arrhythmias in Koch’s Triangle

**Figure 4:**
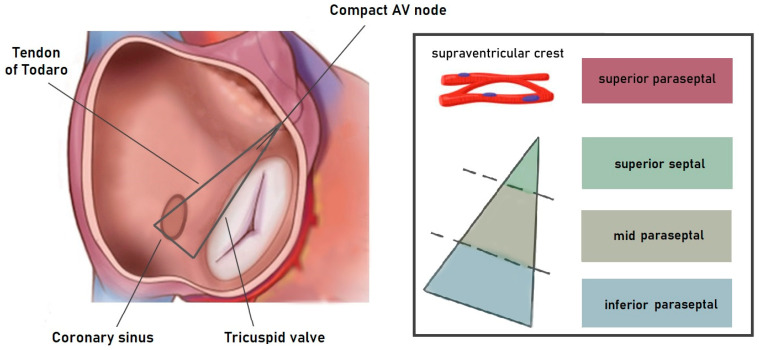
A schematic graphical representation of the area proximal to the hinge of the septal leaflet of the tricuspid valve. The Koch’s triangle corresponds to the atrial aspect of the anatomic mid-paraseptal region. The upper part of the paraseptal area (superior paraseptal) is located between the supraventricular crest and the upper vestibular section of the right atrioventricular junction, adjacent to the right coronary sinus of the aorta, and antero-superior to the membranous septum. It is part of the right atrioventricular groove but is not considered part of the septum itself [38]. The superior septal area corresponds to the membranous septum, which is the only true anatomical septum. The mid-paraseptal region is the upper portion of the inferior pyramidal space, positioned between the right atrium and left ventricle. It forms the floor of Koch’s triangle, with the atrioventricular node on its top [38]. The inferior paraseptal region corresponds to the septal isthmus, which lies between the opening of the coronary sinus and the septal attachment of the tricuspid valve’s septal leaflet. It represents the lower part of the inferior pyramidal space, between the anterior wall of the coronary sinus opening and the left ventricle [38].

Koch’s triangle and pyramid are sites of numerous arrhythmic substrates, ranging from accessory AV pathways to foci of ventricular and atrial arrhythmias. Particularly relevant are the substrates in the parahisian septal and paraseptal areas. The triangle of Koch represents the atrial aspect of the anatomical area proximal to the hinge of the septal leaflet of the tricuspid valve, previously incorrectly called the AV septum, which can be divided into superior paraseptal, superior septal, mid paraseptal, and inferior paraseptal regions [37,38] (Figure 4).

### 5.1. Parahisian Accessory Pathways

**Figure 5:**
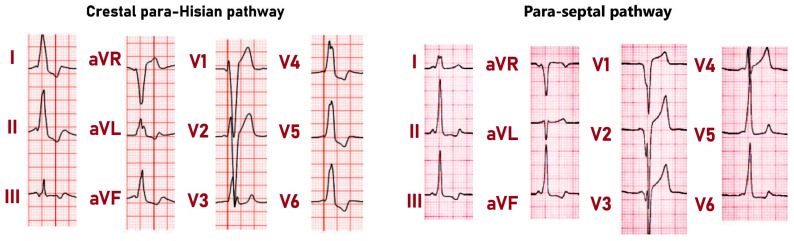
On the left side: A parahisian accessory pathway with ventricular insertion near the crest, showing a delta wave in lead III that is initially negative during sinus rhythm. Positive delta waves are seen in leads I, II, aVF, V4, V5, and V6. The fusion of the P-wave and delta wave is present in leads I, II, and aVF. On the right side: A parahisian septal accessory pathway. In lead I, there is an evident fusion of the P wave with the delta wave. The QRS complexes, along with their initial delta waves, appear positive in leads I, II, III, and aVF. In leads V1 and V2, the QRS complexes display a QS pattern with notching seen in the descending part of the S wave.

The superior area of this vestibular septal tricuspidal region is also parahisian and is the location of the proximal insertion of many parahisian pathways. These pathways insert distally into the paraseptal components of the supraventricular crest or the summit of the muscular ventricular septum after crossing the membranous septum, thereby forming true septal parahisian accessory pathways [39]. This parahisian area includes the superior apex of the Koch triangle, containing the compact AV node, the membranous septum, the penetrating bundle, and the paraseptal location of the supraventricular crest. Crestal pathways insert distally at the paraseptal segment of the supraventricular crest, and are also parahisian, as they cross the AV junction near the His bundle. Meanwhile, pathways that cross the membranous septum to insert into the summit of the interventricular septum are parahisian and truly septal, because they cross the membranous septum without exiting the heart. Due to the different locations of the ventricular insertion, paraseptal and septal parahisian accessory pathways display two distinct ECG patterns during anterograde conduction. In crestal parahisian pathways, the QRS in V1 shows an rS morphology with a broad low-voltage initial r wave and a duration of 140 ms or more, whereas in septal parahisian pathways, the QRS exhibits a QS morphology in V1–V2 with a QRS duration within 120 ms (Figure 5).

According to a recent proposal by Anderson et al. [4], accessory pathways previously termed anteroseptal are actually superior and septal, and should be defined as parahisian because at least a segment of their course at the AV junction is close to the normal AV conduction axis. The crestal paraseptal pathways can be accessed from either the right side of the heart or the non-coronary or right aortic sinus of Valsalva, while the septal parahisian pathways can only be ablated from the right side of the heart. When ablating these pathways, it is preferable to select ablation sites farther from the His bundle, where only a small far-field His electrogram is recorded in the beats conducted via the normal conduction system (during orthodromic tachycardia or during programmed atrial stimulation). However, the direct recording of AV nodal potentials may help to avoid injury to the compact AV node.

### 5.2. Parahisian Atrial Tachicardias

**Figure 6:**
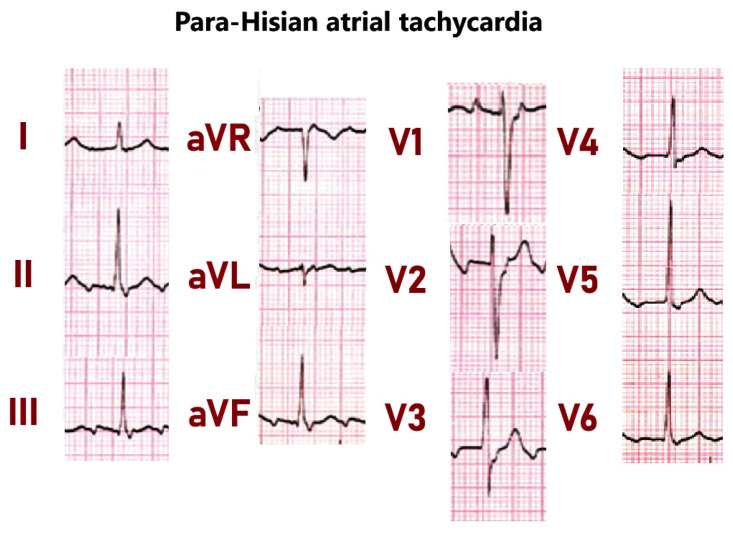
Parahisian atrial tachicardia. Note the negative–positive P-wave in lead V1 and the negative–positive P-wave in lead III.

Parahisian region is also a source of atrial tachycardias. In these tachycardias, the earliest atrial activation is typically recorded within 1 cm of the recorded His signal, and the P wave morphology is mostly negative/positive in lead V1, positive or negative/positive in leads I and aVL, with variable morphologies in the inferior leads and narrow P wave width [40] (Figure 6). Parahisian ATs are sensitive to adenosine and verapamil, which support a mechanism of triggered activity [41]. In these tachycardias, ablation at the non-coronary sinus of Valsalva is the preferred ablation site, due to the lower risk of conduction system damage compared to ablation in the right atrium. Although His bundle potentials are recorded at the junction of the NC and the right coronary sinus of Valsalva, a His potential is generally not recorded more posteriorly in the NC sinus, where ablation is safe and effective.

This approach can also be effective when the activation time in the NCC is not the earliest compared to the parahisian atrial sites. In this regard, the non-coronary sinus of Valsalva provides a vantage point far enough from the conduction system [42]. When this approach is not effective, ablation in the parahisian right atrium, at sites where a low-voltage far-field His electrogram is recorded, is necessary, which carries a higher risk of conduction system damage, junctional rhythm induction, and/or transient AH prolongation. Again, if ablation is performed in the right paraseptal and parahisian region, recording AV nodal potential may be useful to avoid sites where a compact AV node potential is recorded.

### 5.3. Parahisian PVC/VT

**Figure 7:**
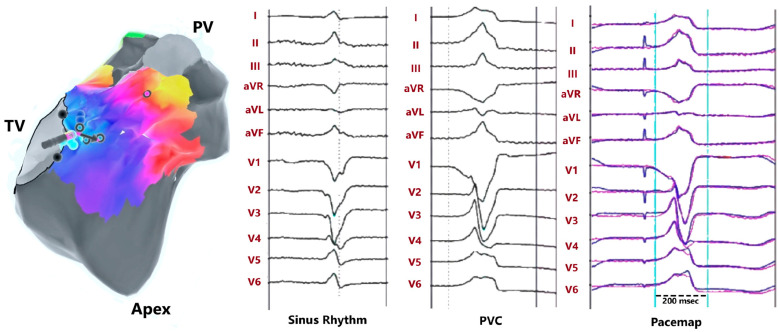
Mapping a PVC from the right parahisian region. The earliest activation was recorded under His electrogram, marked by blue/cyan dots. Tracings show recordings during ablation in sinus rhythm, PVC, and during pacemapping. PV = pulmonary valve; TV = tricuspid valve.

Also, ventricular arrhythmias (VAs) in the form of both ventricular tachycardias and premature ventricular contractions (PVCs) can originate in the vicinity of the His bundle region and are thus defined as parahisian when the earliest activation is recorded in the presence of a His potential or within a 10 mm distance from the His recording site [43]. Regarding PVC, the parahisian region corresponds to the membranous septum and the crest of the muscular septum when the His bundle bifurcates into the left and right bundle branches [44]. The membranous septum from the right side is covered by the septal leaflet of the tricuspid valve, which divides it into an atrioventricular and an interventricular component, while from the left side it is located below the junction of the right and non-coronary sinus of Valsalva. The position of the parahisian zone accounts for the distinct ECG features of parahisian VA, namely a left bundle branch block morphology with a QS pattern in V1 and variable precordial transition (usually at V2–V3), a relatively narrow QRS, an inferior axis with a taller R wave in lead II than in lead III, or an inferior lead discordance with a positive lead II and a negative lead III (Figure 7).

Mapping should cover all the anatomical structures contiguous with the parahisian area, including the RV septum, the right and the non-coronary sinus of Valsalva, and the left ventricular septum below the aortic valve. An earlier activation time can be found in these structures, making the ablation more effective and safe. Moreover, even when the activation time in these anatomical structures is later than that recorded by the hisian catheter, ablation at these sites remains safer and still effective. Therefore, the mapping of the right and non-coronary sinus of Valsalva should always be performed even when right and left ventricular mapping shows the earliest activation at the His bundle recording site. As previously reported, recent histological studies and a virtual dissection of a magnetic resonance dataset have shown that the concept of Koch’s triangle should be reconsidered and defined as Koch’s pyramid, according to its relationship with the pyramid space and the inferoseptal recess of the left ventricle. The inferior extent of the recess is positioned in strict contact with the apex of Koch’s pyramid, with the KT forming the right triangular face of the pyramid; this relationship allows the atrioventricular conduction axis to transition directly from the atrial walls to the crest of the muscular ventricular septum. The inferoseptal recess may also be the source of VA, which can be treated successfully by endocardial catheter ablation from the inferoseptal recess [45] or, concerning the anatomical relationships described above, from the inferomedial right atrium near Koch’s triangle [46].

## 6. New Perspective in Cardiac Imaging: Hierarchical Phase-Contrast Tomography

Modern scanning equipment is equipped with ever-increasing detectors, enabling a level of detail acquisition that was unattainable until a few years ago. However, they are unable to provide details about the structural changes in the heart through length scales necessary for understanding the underlying pathophysiology of diseases. In the latest years, the Hierarchical Phase-Contrast Tomography (HiP-CT) technique has emerged as a powerful tool for the multidimensional analysis of the adult human heart, providing unprecedented insights into both normal and diseased states, with a non-destructive approach that allows for the examination of structural normalcy and abnormalities at scales ranging from 20 to 2.2 µm per voxel [47]. It has demonstrated its capability to unveil novel details of the cardiac conduction system, particularly focusing on the sinoatrial (SA) and atrioventricular (AV) nodes. In healthy hearts, HiP-CT revealed the SA nodal artery’s lateral origin from the circumflex coronary artery. The SA node cells intricately followed the artery’s course, forming connections with the atrial myocardium and the paranodal area. Conversely, in diseased hearts, the SA nodal artery arose laterally from the stented region of the right coronary artery, but with altered cell morphology and attenuated connections to the atrial myocardium. In addition, the AV node’s location, traced through the AV nodal artery, showed changes in the diseased heart, separated and infiltrated by fatty tissue. Connections to the atrial septum and ventricular septum were blunted due to fatty infiltration. HiP-CT successfully visualized the course of the penetrating bundle (of His) and the bundle branches, providing insights into the proximal left and right bundle branches [47]. The perspectives of its use are manifold, and in the study of Koch’s pyramid arrhythmias, it is limited not only to the anatomical definition of structures connected to the AV node but also to the definition of the structural changes associated with diseased hearts, which are responsible for the occurrence and maintenance of Koch’s pyramid arrhythmias. However, the imaging was performed ex vivo and in autopsy studies, which may not fully reflect the dynamic and muscle behavior of the organ in vivo. The main limitation preventing in vivo scanning with the HiP-CT approach is the high radiation dose associated with synchrotron imaging.

## 7. Discussion

The challenges in defining the AVNRT circuit in humans persist due to contrasting hypotheses about its composition, location, and the size of the components involved. One key challenge is the variability between animal and human hearts, with significant differences in structures like the PB and compact AVN. Anatomical variations, such as the absence of the infero-septal recess in certain species, emphasize the need for cautious extrapolation. Knowing the variability of the conduction system is crucial, not only to reconstruct the circuits involved in cardiac arrhythmias but also to prevent potential iatrogenic injury in interventional procedures. With the increase in structural procedures, conduction issues post-transcatheter aortic valve replacement have become more common. In this context, the position and relationship between the depth of the infero-septal recess and the angle of the ventricular septum is of particular importance to prevent conduction problems during valve implantation [48], making it necessary to redefine the right fibrous trigone and the central fibrous body [49]. Efforts to map the AVNRT circuit face hurdles in recording AV nodal potential due to low tissue mass, slow conduction velocity, and technical limitations. Attempts to improve catheter technology and automated mapping systems offer promise for capturing crucial signals in a clinical setting [50].

## 8. Conclusions

In conclusion, our review embarks on a journey through the intricate history and evolving landscape of cardiac conduction research, with a specific focus on the AV conduction axis and its implications in AVNRT. In a hundred years, our understanding of the Kock pyramid has evolved, and with it, our anatomical, histological, and physiological knowledge has improved. Structures such as the compact node, lower nodal bundle, and various extensions exhibit diverse shapes, dimensions, and histological characteristics, influencing their electrophysiological properties. The relevance of these new insights lies in their potential to enhance our understanding of this complex anatomical region, However, challenges persist in defining the AVNRT circuit in humans, highlighting the need for a multidimensional approach that incorporates technological advancements, anatomical understanding, and clinical validation through human mapping studies to improve the safety and efficacy of ablation procedures and optimize pacing lead implantation.

## Data Availability

Not applicable.
